# Development and Validation of Parental Support Scale of Children’s Sports Training in China: Socio-Ecological Approach

**DOI:** 10.3389/fpsyg.2022.922282

**Published:** 2022-06-16

**Authors:** Nan Zheng, Ye Yang

**Affiliations:** ^1^School of Physical Education and Sport Training, Shanghai University of Sport, Shanghai, China; ^2^School of Continuing Education, Shanghai University of Sport, Shanghai, China

**Keywords:** scale development, parent support, children, sports training, socio-ecological

## Abstract

**Objectives:**

To promote Chinese children participation in sports training and acquisition of sports skills, we combined a social ecological research framework with parental support to develop the Parental Support for Children’s Sports Training Scale (PSCSTS).

**Methods:**

The scale was initially developed based on literature review, group interviews, and expert evaluations. A complete reliability and validity test was conducted on 1,594 parents of primary and secondary schools in Shanghai.

**Results:**

The PSCSTS has 37 questions, and exploratory factor analysis has formed 10 factors: policy support, parents’ financial support, community support, media support, sports development, school activity participation, parents’ exercise habits, training quality support, development of school sports activities, learning skills. The higher-order models constructed by validated factor analysis fit well (*χ*^2^/*df* = 2.130, RMSEA = 0.038, SRMR = 0.042, GFI = 0.911, CFI = 0.928). The Parents of children of different sexes have measurement invariance in the PSCSTS.

**Conclusion:**

The PSCSTS developed in this study based on a social-ecological framework has high reliability and validity, and can be used as a comprehensive measure of parental support for their children’s sports training.

## Introduction

A better level of sports skills is an important factor in promoting individuals to maintain lifelong sports behavior. The current situation of Chinese children’s mastery of sports skills is not promising; they are still not proficient in a sports skill after years of physical education courses, which is an important reason for the lack of physical activity and the increase of obesity among Chinese children (Mao, 2019). Aside from physical education classes, various forms of sports training (school sports clubs, social sports training institutions, etc.) are important ways to help children’s master sports skills. Children’s participation in such organized sports training activities may require more support from their parents.

Parental support is an important factor in children’s successful participation in organized sports training (Rittsteiger et al., 2021). Parental encouragement, behavioral support, and financial support are important factors in maintaining youth participation in a sport over time (Desroches et al., 2022). Existing research has measured parental support for their children’s physical activity more along one dimension or a few items (Kirk et al., 1997; King et al., 2008; Hosseini et al., 2013). But parental support may include a variety of different behaviors, such as cheering on the sidelines, providing training funds, providing transportation, etc. (Knight et al., 2016). Thus, measuring parental support for sports training with a single question or a small number of items, while convenient, may not be sufficiently valid. In addition, the simple form of the measure only captures differences in overall parental support and does not reflect differences in different aspects of parental support.

Currently available instruments for measuring social support in sport such as the Perceived Available Support in Sport Questionnaire (PASS-Q; Freeman et al., 2011), and the Social Support Survey (SSS; Rees et al., 2000). Although these scales have a multidimensional structure, they are more suitable for evaluating the social support received by sport participants rather than how to promote supporter support. Specifically for example, the four-dimensional structure of the PASS-Q (emotional, Esteem, information, and tangible) can effectively evaluate the degree of support received by exercise participants on the corresponding dimensions, but cannot provide suggestions for improving supporter support. Therefore, we believe there is a need to develop a measurement tool to measure Chinese parents’ total support for their children’s participation in sports training and where this support originates.

Parents’ support for their children’s participation in sports training comes from a variety of sources. At the level of individual motivation, participation in sports contributes to mental health, emotional development, and physical health (Biddle et al., 2011; Warburton and Bredin, 2017; Brière et al., 2020), organized sports activities are more beneficial for children to acquire sports skills (Vandorpe et al., 2012), and some parents may see supporting their children’s participation in sports training activities as an investment in the hope that their children will receive scholarships or become professional athletes in the future (Bean et al., 2014). Beyond the individual level, family support, socioeconomic status, and parental sports behaviors may influence children sports behaviors (Eime et al., 2013; Brown et al., 2020), the physical activity climate in the community may influence children physical activity levels (Cohen et al., 2017), and even more macroscopically, mass media and policies may influence children physical activity (Pate et al., 2011). It can be seen that parental support can come from several levels. Clearly, a comprehensive theoretical framework is needed to identify those influences that can drive parents to support their children’s participation in sports training.

In recent years, social ecology has provided a new perspective for sports scholars to analyze the influencing factors of children sports behavior. This theory has a certain superiority over health belief theory and planned behavior theory, which intervene in individual sports behavior from internal factors, and social ecology emphasizes the core influence of the environment on sports behavior change, which provides ideas for establishing a long-term mechanism of children sports behavior intervention. The ecological model was first proposed by Professor Bronfenbrenner (1979) who believed that the interaction between individual behavior and the environment should be understood at different levels, such as micro, meso, and macro, and then Mcleroy et al. (1988) applied the ecological model to the field of health promotion by analyzing and developing health promotion programs at five levels: intrinsic factors, interpersonal, institutional, community, and policy.

The social ecology model is a valid analytical model for promoting individual behavior. The model is a multilevel structure, and influences that can promote children’s physical activity behaviors exist at different levels (e.g., family level, school level, community level, etc.). Interventions at multiple levels are more effective than interventions at only the psychological level of the individual, so it is more effective to develop behavioral intervention programs based on multiple levels of influences. This model is highly adaptable and can be applied to interventions for other individuals who do not use sports behaviors (Sallis et al., 2015). In addition, the different socio-ecological levels do not exist in isolation from each other. The policy level may influence children’s sports behavior by influencing the school level (Langille and Rodgers, 2010). The socio-ecological model can also be applied to health behavior interventions for adults, confirming that the environmental level and policy level can promote physical activity behavior and healthy eating among rural community residents (Barnidge et al., 2013). Therefore, we believe that it is reasonable and innovative to apply the social ecological model to the analysis of the division of parental support factors for their children’s participation in sports training.

In summary, we believe that the multilevel structure of the social ecological model is an effective analytical model for exploring parental support factors. Therefore, we innovatively combined the social ecological model with parental support factors and combined it with the Chinese cultural context to develop the Parental Support for Children’s Sports Training Scale (PSCSTS). It aims to effectively and comprehensively measure Chinese parents’ support for their children’s participation in sports training.

## Materials and Methods

### Scale Construction

The PSCSTS was constructed mainly through literature review, interviews, and expert evaluation.

The specific question items at each level were primarily based on the social-ecological scale of children physical activity developed in recent years in the Chinese cultural context by Huang and Zhang (2020), Xiang (2019), and Chen and Sun (2014), and the expressions were modified according to the context of sports training while ensuring that the core concepts of the question items remained unchanged.

We interviewed 21 parents whose children were participating in sports training. The interviews were conducted on a telephone basis, and we stated that all information from the conversations would be used for academic research only, no personal information would be disclosed, all subjects participated voluntarily, and the interview time was limited to 5 min. All information is organized, categorized and used to expand the question items. It is worth noting that during preliminary discussions with the parents of children, some of them believed that “it does not matter where to train, but it mostly depends on whether the coach is professional enough” and that “the conditions of school venues and facilities are poor, so they participate in off-campus training institutions.” In order to fully reflect the characteristics of reflecting children physical activity, this study designed the training quality support dimension in the ecological model.

Finally, we sent all the questions to six professors at Shanghai University of Sport, and the questions and categories that were considered unreasonable would be deleted.

The initial scale had seven dimensions in all. There were 16 items in the internal motivation dimension, 17 items in the family support dimension, 6 items in the training quality support dimension, 9 items in the school support dimension, 7 items in the community support dimension, 7 items in the policy support dimension, and 7 items in the media support dimension, for a total of 69 items, using a 5-point Likert scale, including “totally disagree,” “disagree,” “average,” “agree” and “totally agree” with scores of 1, 2, 3, 4, and 5, respectively.

### Calibration Tools

The Physical Activity Rating Scale (PARS-3; Liang, 1994) was used to evaluate parents’ physical activity behaviors using three topics: single duration, frequency, and intensity of participation in physical activity activities in the last 3 months, scored on a five-point Likert scale, and considered to have good school standard association validity if there was a positive correlation between support scores for each dimension and parental exercise behavior scores.

### Distribution and Collection of Questionnaires

We conducted the survey from October to December 2021. We selected 16 schools in 16 districts in Shanghai, including 12 elementary school and four secondary schools, making the sample as representative of Shanghai as possible. The survey was conducted among the parents of some students in these schools. In the preparation phase of the survey, the initial questionnaire was created electronically and completed by online responses. A teacher from each school was contacted to assist in the delivery of the study questionnaire, and the QR code and web link to the electronic questionnaire were sent by the teacher to a parent communication group.

All surveys were voluntary, and respondents read the informed consent form before starting to respond. We declare that all information will be kept confidential and can be withdrawn at any time during the filling process. In addition, to ensure the validity of the questionnaires, the questionnaires were considered invalid if they contained a large number of identical responses, if they were incomplete, or if they were completed too quickly (at least 3 s for each item).

The final sample consisted of 1,594 parents of students, and we called back 25 parents of students, 23 of whom were willing to be retested at 15-day intervals. The age range of the respondents’ children was between 6 and 17 years old, Media = 9.00, Mean (*M*) = 9.77, and SD = 2.64. The gender of the survey respondents’ children was 46.55% male (*n* = 742), 33.06% female (*n* = 527), and 20.39% both male and female (*n* = 325).

The 1,594 samples will be randomly divided into two separate groups in SPSS, Sample A (*N* = 797) and Sample B (*N* = 797), with Sample A undergoing exploratory factor analysis and Sample B undergoing confirmatory factor analysis. The recommended requirement for conducting factor analysis is that the sample size be greater than five times the number of items (Hair et al., 2011), and the initial number of scale items in this study was 69, and both Samples A and B were able to meet the recommended criteria.

### Statistical Analysis

In exploratory factor analysis (EFA), this study used principal component analysis to extract factors with eigenvalues greater than 1, the optimal oblique intersection method was used for factor rotation, and the existence of a certain correlation among the factors was more in line with theoretical reality.

In the confirmatory factor analysis (CFA), we examined the common method variance (CMV) and measurement invariance of the model. The final attempt to construct a higher-order model based on the first-order model.

The analysis was performed using SPSS 26.0 with AMOS 24.0.

## Results

In SPSS, we randomly selected 50% of the 1,594 samples as Sample A (*n* = 797) for item analysis and exploratory factor analysis (EFA), and the remaining 50% as Sample B (*n* = 797) for confirmatory factor analysis (CFA).

### Exploratory Factor Analysis

After removing two polygraph questions, the remaining 69 questions were included in the factor analysis and the Kaiser-Meyer-Olkin (KMO) and Bartlett’s tests were as follows.

Table 1 shows that the KMO value reached 0.929, representing an excellent score, indicating the presence of partial correlation between the variables (Kaiser, 1974). Bartlett’s test reached the significance level, indicating that linear combinations existed and variables within the population correlation matrix were uncorrelated (Watson, 2017). This indicates that the data is suitable for EFA.

**Table 1:** KMO and Bartlett’s test.

Number of items	Kaiser-Meyer-Olkin	Bartlett	*χ* ^2^	*df*	Sig.
69	0.929	0.000	20233.580	2,346	0.000

The extraction method in EFA in SPSS was the principal component method, and the rotation method was Promax. The criteria for removing items in EFA are (1) factor loadings below 0.45, (2) the number of items under a single dimension less than 3, (3) cross-loadings greater than 0.40, and (4) items that cannot be explained by theory. Only one question item was deleted each time, and then EFA was performed. After several operations, 13 question items were finally deleted and 56 question items were retained, with 12 factors with eigenvalues greater than 1, which cumulatively explained 55.655% of the total variance, and the factor loadings of each question item were between 0.488 and 0.839. In the remaining 56 items, the Skewness of each variable was less than 2 and the Kurtosis was less than 5, so each variable can be considered to follow a normal distribution (Curran et al., 1996), as shown in Table 2.

**Table 2:** Descriptive statistics and factor loading of items.

Items	Mean	SD	Skewness	Kurtosis	Factor loading
Q58	3.536	0.933	−0.407	−0.124	0.809
Q59	3.184	1.022	−0.121	−0.485	0.797
Q60	3.986	0.826	−0.726	0.572	0.606
Q61	3.699	0.972	−0.463	−0.198	0.758
Q62	3.459	1.039	−0.480	−0.258	0.744
Q63	3.657	1.031	−0.589	−0.221	0.728
Q64	3.380	1.075	−0.259	−0.534	0.828
Q50	3.980	0.781	−0.520	0.093	0.595
Q52	3.346	1.029	−0.197	−0.525	0.708
Q53	2.974	1.136	0.036	−0.759	0.763
Q54	2.908	1.103	0.125	−0.679	0.761
Q55	3.546	1.001	−0.616	0.012	0.768
Q56	3.567	0.978	−0.403	−0.207	0.771
Q11	2.947	1.044	0.099	−0.380	0.710
Q13	2.349	1.063	0.673	−0.037	0.749
Q14	2.780	1.081	0.038	−0.667	0.816
Q15	3.069	1.092	−0.096	−0.592	0.760
Q16	3.862	0.848	−0.662	0.502	0.597
Q65	3.912	0.760	−0.574	0.411	0.681
Q66	3.817	0.994	−0.695	0.070	0.617
Q67	3.353	1.083	−0.253	−0.561	0.669
Q68	3.706	0.969	−0.722	0.285	0.737
Q69	3.838	0.822	−0.563	0.382	0.635
Q70	3.858	0.859	−0.631	0.434	0.598
Q41	4.034	0.698	−0.401	0.274	0.746
Q42	4.325	0.824	−1.258	1.576	0.764
Q45	3.846	0.904	−0.541	0.056	0.570
Q47	4.099	0.809	−0.867	0.942	0.733
Q17	3.746	0.896	−0.589	0.153	0.633
Q18	3.541	1.005	−0.354	−0.456	0.773
Q19	4.271	0.698	−0.782	0.811	0.601
Q22	3.734	0.967	−0.502	−0.224	0.783
Q23	3.844	0.945	−0.626	0.027	0.644
Q36	4.327	0.809	−1.205	1.349	0.720
Q37	4.286	0.777	−1.189	2.137	0.718
Q38	4.276	0.758	−0.959	1.032	0.649
Q40	4.523	0.609	−1.649	3.620	0.677
Q1	4.641	0.560	−1.628	4.117	0.690
Q3	4.463	0.628	−0.866	0.499	0.586
Q5	4.592	0.574	−1.303	2.246	0.642
Q7	4.408	0.697	−1.131	1.601	0.634
Q9	4.349	0.720	−1.038	1.401	0.516
Q25	3.524	1.071	−0.418	−0.586	0.759
Q26	3.344	1.130	−0.358	−0.712	0.803
Q27	3.412	1.128	−0.411	−0.562	0.782
Q2	4.168	0.726	−0.643	0.533	0.622
Q4	4.077	0.831	−0.670	0.244	0.735
Q6	4.156	0.752	−0.619	0.141	0.547
Q8	3.858	0.893	−0.408	−0.339	0.711
Q43	3.737	0.892	−0.269	−0.420	0.704
Q46	3.906	0.867	−0.479	−0.239	0.674
Q48	3.897	0.849	−0.396	−0.236	0.763
Q29	3.345	1.107	−0.290	−0.586	0.593
Q30	3.512	1.034	−0.401	−0.416	0.694
Q31	4.072	0.832	−0.869	0.931	0.626
Q33	3.577	1.120	−0.452	−0.505	0.714

### Confirmatory Factor Analysis

The remaining 56 question items were included in AMOS for first-order oblique CFA using the maximum likelihood estimation. Before the CFA, we used an online calculator to calculate the required sample size for the CFA, using a date of May 4, 2022 (Soper, 2022). We entered the results given by the EFA (12 latent variables and 56 observed variables) into the calculator, with the Anticipated effect size set to 0.3 and the desired statistical power level designed to 0.8. The probability level is designed to be 0.05. The results suggest that the minimum sample for the model structure is 89, and the recommended minimum sample size is 200, so Sample B (*n* = 797) can be subjected to CFA.

Referring to the suggestion of Hooper et al. (2008), the rules for CFA include: (1) rejecting inter-residual correlations and eliminating questions with high residual MI correction indices; (2) standardized factor loadings < 0.50; and (3) the number of variables within dimensions < 3. A total of 19 questions were removed in CFA, of which Q3 was eliminated due to low factor loadings, Q1 and Q9 were difficult to form a dimension, so they were both removed. The final first-order oblique model includes 37 observed variables with a total of 10 dimensions. We used *χ*^2^/*df*, RMSEA, SRMR, GFI, and CFI to evaluate the fit of this model. *χ*^2^/*df* should be less than 5, RMSEA should be less than 0.1, SRMR should be less than 0.05, GFI should be greater than 0.8, and CFI should be greater than 0.9 (Bentler, 1990; Browne and Cudeck, 1993; Seyal et al., 2002; Schumacker and Lomax, 2004; see Table 3).

**Table 3:** Fit index of first-order oblique factor model.

Model	*χ* ^2^	*χ*^2^/*df*	RMSEA	SRMR	GFI	CFI
First-order oblique factor model	1198.167	2.052	0.036	0.038	0.921	0.937

The fit index of the first-order oblique factor model in this study were able to meet the recommended criteria.

The remaining 10 dimensions were named by the common characteristics of the question items within each dimension, see Table 4.

**Table 4:** Items and dimensions of PSCSTS (translated from the Chinese version).

Items	Factor
59: Familiar with children sports policies in recent years	Policy support
60: Enactment of children sports policies can promote my support for my children’s participation in sports training	
61: Interested in children sports policies	
62: Know where to check children sports policies	
63: Often see children’ sports policies promoted	
64: Often check children sports policies voluntarily	
52: Sports activities are well built near my community	Community support
53: Children sports activities are often organized near my community	
55: Children often exercise near my community	
56: Good atmosphere for physical exercise near the community	
25: I have a fixed activity content for each sports meeting	Parents’ exercise habits
26: I have a fixed time for each sports meeting	
27: I have a fixed exercise area for each sports meeting	
17: I support my child to go to a paid gym to exercise	Parents’ financial support
19: I support my child to buy sports equipment	
22: I am able to provide my child with various fitness and recreational protection	
23: I support my child to spend money on sports training	
11: Participation in training is good for getting competition rankings	Sports development
13: Participation in training is good for realizing parents’ sports dreams	
14: Participation in training facilitates entry into professional sports teams	
2: Children’s participation in sports training facilitates the acquisition of a sports skill	Learning skills
4: Mastery of a sports skill is important	
8: Mastery of a sports skill is indispensable for lifelong growth	
43: The school has abundant after-school sports competitions	Development of school sports activities
46: The school’s sports games are well developed	
48: The school often conducts various sports competitions and training activities	
41: Children like to play sports at school	School activity participation
42: Children like to take physical education classes	
45: Children like physical education teachers	
47: Children like to participate in various sports activities at school	
36: I value the training quality of the training institution	Training quality support
37: I value the professional knowledge of the trainer	
38: I value the professional skills of the trainer	
65: Broadcast of exciting events can increase my support for my children’s participation in training	Media support
66: My children will want to go to sports training if they have a sports star they admire	
67: I have a favorite sports star that can increase my support for my children’s participation in training	
68: High profile media coverage of athletes is good for increasing my support	

### Model Structural Validity

#### Reliability and Convergent Validity

In this study, average variance extracted (AVE) was used to evaluate the convergent validity of the dimensions, and composite reliability (CR) were used to evaluate the consistency of the dimensions. The recommended value for CR is greater than 0.7 (Raykov, 1997; Hair et al., 2019), AVE ≥ 0.50 is ideal (at this time the average of standardized factor loadings within the dimension should be greater than 0.71), 0.50 > AVE ≥ 0.36 is acceptable (at this time the average of standardized factor loadings within the dimension is 0.71 ~ 0.60; Fornell and Larcker, 1981).

According to Table 5, the standardized factor loadings of all retained question items in this study’s CFA were greater than 0.50 (*p* < 0.001), the CR values of each dimension were 0.640–0.861, with most dimensions able to reach the recommended values, except for LS (CR = 0.658) and TQS (CR = 0.641), which were slightly below the recommended values. The AVE values for each dimension ranged from 0.373 to 0.511, with lower AVE values for some dimensions, probably due to the wide range of ecological factors that make it difficult to have high internal consistency, but all met acceptable criteria. This suggests that the first-order oblique intersection CFA in this study has strong convergent validity and reliability. This indicates that the convergence validity and reliability of the first-order oblique CFA in this study can be achieved.

**Table 5:** Table of reliability and convergent validity.

Variables	Items	S.E.	*T*-value	*P*	Estimate	SMC	CR	AVE
PS	Q59				0.755	0.570	0.860	0.509
	Q60	0.040	14.944	^***^	0.547	0.299		
	Q61	0.045	18.680	^***^	0.675	0.456		
	Q62	0.051	20.308	^***^	0.729	0.531		
	Q63	0.051	19.825	^***^	0.713	0.508		
	Q64	0.052	23.269	^***^	0.830	0.689		
CS	Q52				0.612	0.375	0.789	0.484
	Q53	0.090	15.206	^***^	0.725	0.526		
	Q55	0.081	14.901	^***^	0.702	0.493		
	Q56	0.077	15.349	^***^	0.737	0.543		
SD	Q11				0.611	0.373	0.755	0.511
	Q13	0.096	14.597	^***^	0.816	0.666		
	Q14	0.085	14.347	^***^	0.702	0.493		
LS	Q2				0.564	0.318	0.658	0.393
	Q4	0.118	11.092	^***^	0.685	0.469		
	Q8	0.126	10.924	^***^	0.625	0.391		
PEH	Q25				0.770	0.593	0.755	0.508
	Q26	0.058	16.489	^***^	0.721	0.520		
	Q27	0.054	15.318	^***^	0.641	0.411		
PFS	Q17				0.661	0.437	0.755	0.437
	Q19	0.054	13.693	^***^	0.592	0.351		
	Q22	0.076	14.717	^***^	0.649	0.421		
	Q23	0.073	16.005	^***^	0.734	0.539		
DSA	Q43				0.677	0.458	0.736	0.481
	Q46	0.062	14.685	^***^	0.674	0.454		
	Q48	0.065	15.267	^***^	0.729	0.531		
SAP	Q41				0.709	0.503	0.774	0.464
	Q42	0.064	16.582	^***^	0.701	0.491		
	Q45	0.068	13.340	^***^	0.545	0.297		
	Q47	0.070	17.371	^***^	0.752	0.566		
TQS	Q36				0.607	0.368	0.641	0.373
	Q37	0.092	10.998	^***^	0.613	0.376		
	Q38	0.091	10.999	^***^	0.613	0.376		
MS	Q65				0.628	0.394	0.752	0.431
	Q66	0.091	14.275	^***^	0.650	0.423		
	Q67	0.094	14.882	^***^	0.690	0.476		
	Q68	0.081	14.396	^***^	0.657	0.432		

*** *p* < 0.001.

#### Discriminant Validity

The discriminant validity of the model was tested by comparing the square root of AVE of each dimension to the correlation coefficients of other dimensions. If the square root of AVE is greater than the dimension’s correlation coefficient with other dimensions, it means that each dimension in the model has some internal convergent validity while also having some discriminant validity with other dimensions (Fornell and Larcker, 1981).

As shown in Table 6, the square root of AVE for each dimension of the first-order CFA model was greater than the correlations between the other dimensions in this investigation, showing that the model had high validity.

**Table 6:** A list of discriminant validity.

**Factors**	**1**	**2**	**3**	**4**	**5**	**6**	**7**	**8**	**9**	**10**
1. DSA	**0.694**									
2. MS	0.456	**0.657**								
3. TQS	0.267	0.487	**0.611**							
4. SAP	0.555	0.486	0.395	**0.681**						
5. PFS	0.377	0.648	0.499	0.448	**0.661**					
6. PEH	0.403	0.473	0.387	0.316	0.524	**0.713**				
7. LS	0.345	0.389	0.302	0.321	0.354	0.257	**0.627**			
8. SD	0.268	0.357	0.117	0.128	0.283	0.297	0.417	**0.715**		
9. CS	0.532	0.519	0.203	0.381	0.445	0.437	0.278	0.330	**0.696**	
10. PS	0.440	0.651	0.385	0.440	0.571	0.473	0.368	0.406	0.577	**0.713**

The bolded part is the square root of the corresponding dimension AVE.

### Content Validity and Retest Reliability

Content validity refers to the extent to which test items reflect the subject (Devellis and Thorpe, 2021).

Six physical education professors from Shanghai universities of sport were asked to analyze the questionnaire’s content, and all six professors found the material to be acceptable or extremely reasonable in 100% of the cases, which indicated that the content of the questionnaire reflected the subject of the study.

The scale was distributed to 23 parents of primary and secondary school students in Shanghai and retested after an interval of 15 days. The Pearson correlation coefficient of the total score of the two results *r* = 0.875 (*p* < 0.01) indicates good reliability of the retest of the questionnaire and stable results.

### Common Method Bias Test

Common method variance (CMV) refers to some kind of covariance introduced to all question items by the design of the measurement instrument, the selection of the subject population, and the measurement environment in the study of self-statement scales, and is a kind of systematic error (Richardson et al., 2009). The consequences brought about by CMV are called “common method bias” (CMB), which may have a large negative impact on the accuracy of experimental results, and have gradually been taken seriously by scientific researchers in sociology and psychology in recent years.

We used the Unmeasured Latent Method Construct (ULMC) method and the Harman’s Singer-factor test to examine the CMB of this study.

The first-order CFA model was used as the baseline model to measure the CMB of this study using a control unmeasured potential method factor by adding a common method factor as a global factor in AMOS to construct a ULMC model, and if the model fit index improved substantially, it indicates the existence of serious common method bias (Richardson et al., 2009). The fit indices of the baseline model in this study were not significantly improved compared to the ULMC model (ΔRMSEA = 0.007, ΔSRMR = 0.01, ΔGFI = 0.021, ΔCFI = 0.026; Table 7).

**Table 7:** A list of models fit.

Model	*χ* ^2^	*χ*^2^/*df*	RMSEA	SRMR	GFI	CFI	TF^(a)^
Baseline model	1198.167	2.052	0.036	0.038	0.921	0.937	/
ULMC model	906.576	1.657	0.029	0.028	0.942	0.963	/
Second-order model	1262.980	2.098	0.037	0.040	0.915	0.932	0.949
Third-order model	1311.773	2.130	0.038	0.042	0.911	0.928	0.913

a Target coefficient.

Harman’s Singer-factor test was performed in EFA and the results showed that the single factor without rotation explained 21.743% of the variance, which is less than the 50% criterion (Podsakoff and Organ, 1986). Therefore, it was concluded that there was no serious common method bias in this study.

### Calibrated Correlation Validity

The results of Pearson correlation analysis indicated that the scores of each dimension of the children Physical Training Support Scale were positively correlated with the scores of the Parental Physical Activity Behavior Scale, indicating that this questionnaire has high correlation validity (Table 8).

**Table 8:** Correlation between PSCSTS and PARS-3.

Factors	$\bar{x}$ ***±*** *s*	1	2	3	4	5	6	7	8
1. PS	3.541 ± 0.769	1							
2. CS	3.328 ± 0.817	0.473	1						
3. FS	3.541 ± 0.769	0.505	0.390	1					
4. IM	3.339 ± 0.606	0.369	0.324	0.358	1				
5. SS	3.960 ± 0.564	0.444	0.420	0.444	0.314	1			
6. TQS	4.300 ± 0.600	0.269	0.138	0.370	0.171	0.295	1		
7. MS	3.693 ± 0.724	0.522	0.382	0.508	0.338	0.405	0.318	1	
8. PRS-3	3.692 ± 0.863	0.491	0.423	0.564	0.263	0.392	0.255	0.400	1

### Invariance Test for Different Child Genders

We tested for invariance between the sexes of the children using the sample B (*n* = 797) with male children (*n* = 363) versus the sample with female children (*n* = 280). Because *χ*^2^ is susceptible to sample size, this study used RMSEA, SRMR, and CFI to assess measurement equivalence (Cheung and Rensvold, 2002). For testing loading invariance, a change in CFI ≥ −0.010, a change in RMSEA ≥ 0.015, or a change in SRMR ≥ 0.030 was considered not to have invariance. For testing intercept or residual invariance, a CFI change ≥ −0.010, RMSEA change ≥ 0.015, or SRMR ≥ 0.010 is considered not invariant (Chen, 2007). Model 1 is the baseline model with each parameter freely estimated; Model 2 restricts the factor loadings to be equal between groups based on Model 1; Model 3 restricts the covariance to be equal between groups based on Model 2; and Model 4 restricts the residuals to be equal between groups based on Model 3. The results indicated that the changes in ΔRMSEA, ΔSRMR, and ΔCFI were smaller than the cut-off values for each group, as the PSCSTS can be considered to be measurement invariant for parents with children of different genders (Table 9).

**Table 9:** Test for child gender invariance.

	Model fit			Comparison of model *k* and model *k*–1		
	RMSEA	SRMR	CFI	ΔRMSEA	ΔSRMR	ΔCFI
Model 1	0.030	0.055	0.915	–	–	–
Model 2	0.030	0.056	0.916	0.000	0.001	0.001
Model 3	0.030	0.056	0.914	0.000	0.001	−0.001
Model 4	0.030	0.057	0.913	0.000	0.002	−0.002

### Higher-Order Model Building and Evaluation

Higher-order factor modelling requires theoretical interpretability and correlation between factors. In the model construction, the original dimension of “family support” (FS) in the scale design of this study was decomposed into “parent’s exercise habits” and “parent’s financial support” in EFA, and the *r* = 0.524 for both. Consequently, it was considered necessary to construct a second-order factor named “family support” (FS). Similarly, the original “internal motivation” dimension was decomposed into “sports development” and “learning skills” with *r* = 0.417 to construct a second-order factor named “Internal motivation” (IM). The original “school support” dimension was decomposed into “development of school activities” and “school activity participation,” with *r* = 0.555. Therefore, the second-order factor, named “school support” (SS), was constructed, and the first-order 10-factor model was streamlined into a second-order seven-factor model, which is consistent with the assumptions made at the beginning of the scale. And the fit index of this second-order model can meet the recommended criteria (Table 7).

**Figure 1:**
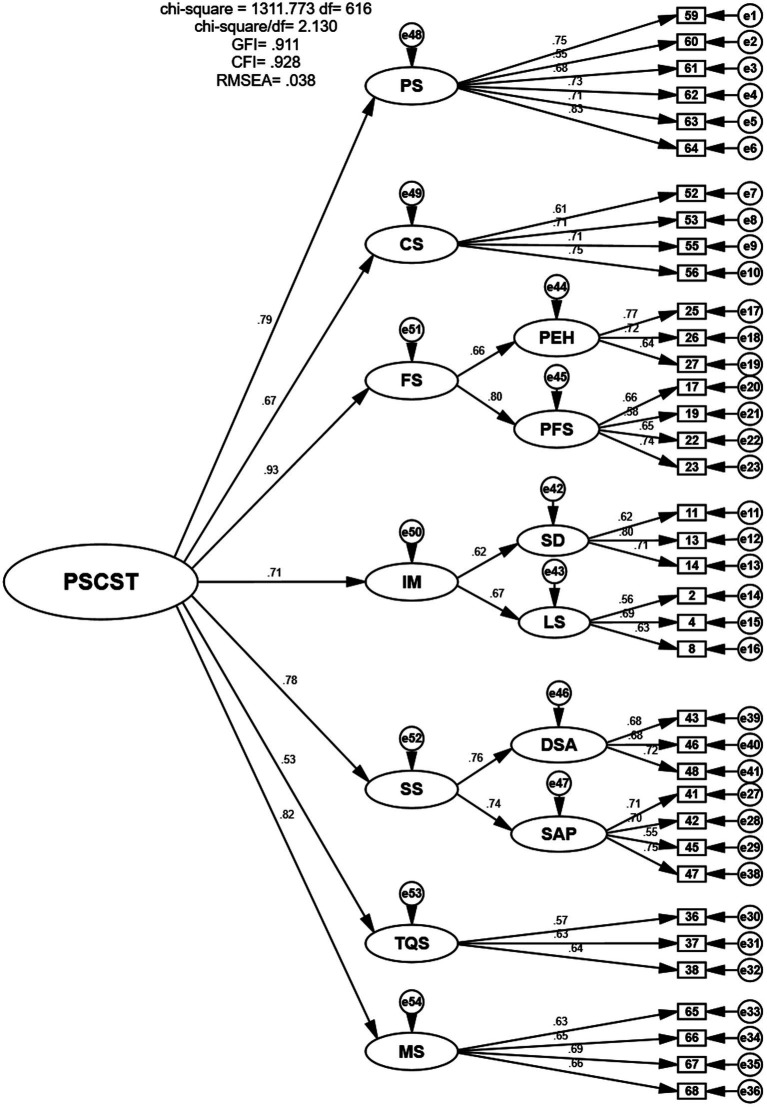
Third-order factor model of parental support for children’s sports training scale.

In addition, theoretically, the second-order factors all belong to the ecological perspective of the influence of children sports training activities, which may be influenced by a common factor, and the dimensions have different degrees of correlation, *r* = 0.200–0.801. The study tried to construct a third-order factor model based on the second-order factor model, and finally named the third-order factor as “parental support for children’s sports training” (PSCST), and the fit index of the third-order model met the recommended criteria (Table 7), and the loadings of the second-order factor on the third-order factor ranged from 0.53 to 0.93 (*p* < 0.001), as shown in Figure 1.

The evaluation method for higher-order model building often uses the target coefficient (Marsh and Hocevar, 1985) to the Chi-square of the first-order model and the higher-order model, and the closer its value to 1, represents the higher-order model is more representative. As shown in Table 8, the target coefficient of the second-order model and first-order model is 0.949 and the target coefficient of the third-order model and first-order model is 0.913. Therefore, following the principles of model parsimony and theoretical interpretability, we consider the third-order factor model to be the best model, as shown in Figure 1.

## Discussion

Parental support for children’s participation in sports training is an important factor in children’s successful participation in sports training, but the complexity of factors influencing this parental support means that measurement needs to be done at multiple levels. This study attempts to integrate a social ecological model with parental support for their children’s sports training, aiming to develop a measurement tool that can comprehensively measure parental support for their children’s training. We initially identified a seven-level structured scale (policy support, community support, family support, internal motivation, school support, media support, and training quality support) through literature compilation and interviews. We explored and validated the internal structure of the model through factor analysis. A higher-order factor model was constructed based on theoretical drivers, while also taking into account model fitting (e.g., RMSEA, CFI, GFI, etc.). There is no serious common method bias in this study and the calibration validity is good. The invariance test further illustrates the applicability of the PSCSTS to parents of gender diverse children. It provides a measurement tool to comprehensively measure Chinese parents’ support for their children’s participation in sports training.

### The Framework of Measurement

Parental Support for Children’s Sports Training Scale is a multi-level structure that includes seven dimensions (internal motivation, family support, school support, community support, training quality support, media support, and policy support). As the systemic concept emphasized by social ecology, these seven dimensions do not exist in isolation from each other, but are interrelated (Langille and Rodgers, 2010). Table 6 demonstrates a degree of positive correlation among the dimensions.

Motivation explains why people think and act the way they do (Weiner, 1992). In the present study, the motivation dimension included mastery of sports skills and sports development. The acquisition of sports skills is an important way to maintain regular physical behavior of children and thus promote physical and mental health. Sports development is also important, as mastery of sports skills can help children achieve scholarships in school or higher earnings as professional athletes (Bean et al., 2014). Therefore, the PSCSTS uses these two motives to measure the internal motivational dimension of parental support.

The family is an important place for children to grow and also to receive education. Good sporting habits of parents are a behavioral model that can increase children’s participation in sporting behavior (Sanz-Arazuri et al., 2012). In addition, financial support from parents is an important factor for children’s participation in sports (Kirk et al., 1997), especially for participation in a sport training (requiring coaching costs, venue costs, equipment costs, etc.). Therefore, we used parental support in action and financial support to represent the family support dimension in PSCSTS.

Schools are the main place where Chinese school-age children participate in physical activities and physical education classes (Guan, 2005). The school support dimension consists of the various types of sports activities (sports competitions and school sports clubs) conducted at school and the children’s sports participation behaviors. In this sense, it covers the subject and object of school physical activity as one of the potential factors to improve parental support.

The community is the primary place where children engage in physical activity outside of school versus at home. A good community sport environment can improve the level of physical activity of individuals (Slater et al., 2010), and the community support dimension of this study includes the construction of community sport facilities and the sporting atmosphere in the neighborhood, which should be one of the sources of parental support for their children’s participation in sport training.

The training quality support dimension was derived from interviews with parents. This dimension cannot be categorized under other socio-ecological dimensions, but training quality is an important influence on parental support for their children’s sports training, so we designed this dimension separately as an important factor reflecting the characteristics of sports training activities. It also reflects the main difference between physical training activity and general physical activity, namely that this activity requires more professional coaching organization and a range of venue facilities to support it.

The media support level mainly includes the influence of the broadcast of sports events and the effect of sports stars. Today’s professional sporting events have a strong appeal to both parents and children. For parents who are keen watchers of sports events, the performance of athletes may be an important factor in promoting support for their children’s sports training. As one interesting Chinese study notes, children who dream of becoming sports stars attend sports training schools (a type of school in China that specializes in sports training) and begin to receive professional sports training (Liu and Li, 2014).

Policy supports mainly refers to the policies issued by the Chinese government in recent years to promote various sports training activities for youth and children (e.g., integration of sports and education policy). Government policies that call for social attention to youth sports activities may directly increase parental support. In addition, policies may increase parental support for youth sport training by influencing school sport training efforts and social sport environments (Langille and Rodgers, 2010), suggesting interactions between different levels of social ecology, but this is beyond the scope of this study and will not be discussed in depth. In summary, policy is also an important factor in increasing parental support. Therefore, in this dimension we use some questions that measure the extent to which parents are concerned about Chinese youth sports policies in recent years to reflect policy support.

### Theoretical Implications

Relative to other similar studies (Langille and Rodgers, 2010; Barnidge et al., 2013; Chen and Sun, 2014; Sallis et al., 2015; Xiang, 2019; Huang and Zhang, 2020), this study further expands the application of social ecology in the field of sport behavior field, proving the adaptability of social ecology theory combined with different subject studies, which also provides a basis for future studies to continue exploring the theoretical implications of social ecology in sport behavior research. On the other hand, we provide a comprehensive research perspective on the study of parental support for children and adolescents’ physical activity. Both in terms of measurement instruments and theoretical research, we argue that the sources of parental support are multidimensional. Analysis, measurement, and intervention of parental support for children should begin at multiple levels, and any single level of intervention or measurement may be one-sided.

In terms of measurement instrument development, it is clear that the PSCSTS is a new measurement instrument that can be used to measure Chinese parents’ support for their children’s sports training, filling a gap in localized measurement instruments. Compared to the PASS-Q (Freeman et al., 2011) and the SSS (Rees et al., 2000), the PSCSTS developed in this study focuses more on the supporter than the supported, placing the supporter in the context and examining the influences that can increase parental support.

## Practical Implications

The PSCSTS, constructed and validated in this study based on a Chinese cultural context, can be used to measure parental support for their children’s participation in sports training in China. The validated seven dimensions can assess the role of each dimension in improving parental support. Government organizations and departments can use this measurement tool to measure the overall level of parental support for sports training in a region, analyze the differences in scores across dimensions, and develop appropriate measures to improve parental support to support various forms of sports training efforts. In addition, a unified measurement tool can be used to compare differences in parental support for sports training among Chinese youth and children in different regions.

## Conclusion

This study further extends the application of social ecology theory and develops an instrument based on social ecology to measure parental support for their children’s training. The results show that the PSCSTS includes 37 items and was validated for content validity, structural validity, calibration validity, and internal consistency reliability. The scale can be used to measure the extent to which parents of Chinese primary and secondary school students support their children’s participation in training, providing a usable tool for government departments and institutions to analyze and intervene in parental support for their children’s sports training.

### Future Research

First, it may be more useful to examine differences in parental support for children’s participation in sports training across family situations, for example, by conducting further research on parents with different incomes, parents with different occupations, parents with different sports habits, and parents living in different regions.

Second, this study set up seven dimensions to reflect parental support in physical training activities, and social ecological theory also emphasizes the existence of interaction between different dimensions, so how the dimensions interact with each other remains to be further revealed by longitudinal studies (e.g., whether such effects further increase parental support through the school level, community level, or media level sometime after the policy is released).

Finally, this study developed a research framework based on the social ecology model, which also further demonstrates the applicability and superiority of this theory for research in the field of sport behavior science, which has yet to be extended to more sport research.

### Limitations

First, this study can only be considered as a preliminary development and validation for PSCSTS. A good measurement tool needs to be developed in continuous practice, and although the model fit index of this study was able to meet the recommended standard, the CR and AVE of some dimensions could only be said to barely meet the acceptable standard, specifically because the social ecology perspective is too broad leading to insufficient internal consistency of some dimensions, or because online questionnaires are more difficult to control errors than paper-and-pencil tests.

Second, the measurement invariance of PSCSTS parental gender was not tested in this study and must be tested in more depth with future parents of different genders to test the applicability of PSCSTS.

Finally, the PSCSTS developed in social ecology is more comprehensive but also longer, which may lead to subjects feeling bored in the process of filling it out. Continued revision of the PSCSTS to make it more concise and focused may be more applicable to different contexts.

## Data Availability Statement

The raw data supporting the conclusions of this article will be made available by the authors, without undue reservation.

## Ethics Statement

Ethical review and approval were not required for the study in accordance with the national legislation and institutional requirements. Written informed consent was not required for this study in accordance with national legislation and institutional requirements.

## Author Contributions

YY and NZ conceived the idea and organized the study. NZ conducted the study, analyzed the data, and wrote the manuscript. YY revised the manuscript. All authors contributed to the article and approved the submitted version.

## Funding

This work was supported by Shanghai Education Science Foundation (Project No. C2-2020028).

## Conflict of Interest

The authors declare that the research was conducted in the absence of any commercial or financial relationships that could be construed as a potential conflict of interest.

## Publisher’s Note

All claims expressed in this article are solely those of the authors and do not necessarily represent those of their affiliated organizations, or those of the publisher, the editors and the reviewers. Any product that may be evaluated in this article, or claim that may be made by its manufacturer, is not guaranteed or endorsed by the publisher.
